# Prevalence and determinants of loneliness among the oldest old living in institutionalized settings: Study findings from a representative survey

**DOI:** 10.1007/s00391-023-02196-x

**Published:** 2023-06-02

**Authors:** André Hajek, Larissa Zwar, Razak M. Gyasi, Benedikt Kretzler, Hans-Helmut König

**Affiliations:** 1 Department of Health Economics and Health Services Research, University Medical Center Hamburg-Eppendorf, Hamburg Center for Health Economics, Martinistr. 52, 20246 Hamburg, Germany; 2 Aging and Development Unit, African Population and Health Research Center, Nairobi, Kenya; 3 National Centre for Naturopathic Medicine, Faculty of Health, Southern Cross University, Lismore, NSW Australia

**Keywords:** 80 and over, Loneliness, Social isolation, Social exclusion, Depression, Institutionalization, 80 Jahre und älter, Einsamkeit, Soziale Isolation, Soziale Ausgrenzung, Depression, Heimunterbringung

## Abstract

**Background:**

There is very limited knowledge regarding the prevalence and determinants of loneliness in oldest old residents of nursing or old age homes.

**Objective:**

To examine the prevalence and determinants of loneliness among the oldest old living in institutionalized settings in Germany.

**Material and methods:**

Data were taken from the representative survey on quality of life and subjective well-being of the very old in North Rhine-Westphalia (NRW80+) including individuals ≥ 80 years living in North Rhine-Westphalia. The study focused on individuals living in institutionalized settings. Sociodemographic, lifestyle-related, and health-related determinants were included in multiple linear regression models.

**Results:**

Approximately 56.6% of the individuals were not lonely, 25.7% and 17.8% of the individuals were moderately and severely lonely, respectively. Regression analyses showed that higher loneliness was associated with being married (β = 0.48, *p* < 0.05), high education (compared to low education, β = 0.46, *p* < 0.05), having a small social network size (β = −0.02, *p* < 0.05), having poor self-rated health (β = −0.25, *p* < 0.05), and more depressive symptoms (β = 0.25, *p* < 0.001).

**Conclusion:**

A significant proportion of the institutionalized oldest old individuals reported moderate or severe loneliness, which underpins the relevance of this topic. Understanding the determinants of loneliness may help to address institutionalized adults aged 80 years and over at risk of loneliness.

## Abstract (de)

**Hintergrund:**

Es gibt nur sehr wenige Erkenntnisse über die Prävalenz und die Determinanten von Einsamkeit bei älteren Bewohner:innen von Pflege- oder Altersheimen.

**Ziel der Arbeit:**

Untersuchung der Prävalenz und der Determinanten von Einsamkeit bei älteren Menschen, die in stationären Einrichtungen in Deutschland leben.

**Material und Methoden:**

Die Daten stammen aus der repräsentativen Studie „Lebensqualität und Wohlbefinden hochaltriger Menschen in NRW (NRW80+)“, die in Nordrhein-Westfalen lebende Personen ≥ 80 Jahre umfasst. Wir haben uns auf Personen konzentriert, die in institutionellen Einrichtungen leben. Soziodemographische, lebensstilbezogene und gesundheitsbezogene Determinanten wurden in multiple lineare Regressionsmodelle einbezogen.

**Ergebnisse:**

Etwa 56,6 % der Personen waren nicht einsam, 25,7 % und 17,8 % der Personen waren mäßig bzw. stark einsam. Die Regressionen zeigten, dass höhere Einsamkeit mit Verheiratetsein (β = 0,48; *p* < 0,05), hoher Bildung (im Vergleich zu niedriger Bildung, β = 0,46; *p* < 0,05), einem kleinen sozialen Netzwerk (β = −0,02; *p* < 0,05), schlechter selbst eingeschätzter Gesundheit (β = −0,25; *p* < 0,05) und mehr depressiven Symptomen (β = 0,25, *p* < 0,001) assoziiert war.

**Diskussion:**

Ein signifikanter Anteil der stationär untergebrachten Personen >80 Jahre berichtete über mäßige oder schwere Einsamkeit – was die Relevanz dieses Themas unterstreicht. Das Verständnis der Determinanten der Einsamkeit kann dazu beitragen, institutionalisierte Erwachsene im Alter von 80 Jahren und darüber, die von Einsamkeit bedroht sind, zu adressieren.

## Brief introduction.

In very late life various adverse life events, such spousal loss commonly occur. Often due to a lack of data availability, individuals living in nursing or old age homes are neglected in research. For example, the prevalence and determinants of loneliness in oldest old residents of nursing or old age homes is largely unknown. Such knowledge is of great relevance to better understand the significance of loneliness among the oldest old residing in institutionalized settings. This in turn is of great importance because loneliness predicts morbidity and premature death.

## Introduction

The number of individuals aged 80 years and older (also called oldest old) is expected to rise considerably in the next 20 or 30 years, not only in Germany, but in many industrial nations [27]. In this age bracket, various critical life events can take place such as immobility, cognitive decline, spousal loss, or institutionalization [15]. Particularly oldest old individuals living in institutionalized settings might experience worse conditions but are often neglected in research (e.g., due to a lack of data availability); however, it can be assumed that the importance of nursing and old age homes will increase as the geographic distance to family members (who could potentially provide informal care) is increasing and particularly the daughters, who have often provided private care in the past, are placing increasing emphasis on their professional careers [18, 29, 35].

One main topic for such institutionalized oldest old individuals may be loneliness [39], referring to a discrepancy between actual and desired social relations [38]. Due to various reasons (such as disconnection from friends and acquaintances [5, 20]), particularly individuals residing in institutionalized settings can feel lonely [45, 52]. This would be in accordance with the theory of the need to belong which was proposed by Baumeister and Leary [3] in the mid-1990s. Overall, this topic is of great importance because loneliness can have adverse health consequences such as morbidity and mortality [17, 37, 50].

As described in a recent systematic review and meta-analysis [13], only some studies investigated the prevalence of loneliness among older individuals living in institutions. These studies often included individuals aged 60 years and over [1, 7, 10, 14, 25, 34, 40, 51], whereas only very few studies [30, 36] exclusively focused on the oldest old individuals who most likely considerably differ from individuals aged 60 years (e.g., in terms of mobility, cognitive impairment or availability of friends and relatives, which could be important for loneliness [52]). Moreover, quantitative evidence from Germany is completely missing. Thus, our aim was to examine the prevalence and determinants of loneliness among the oldest old living in institutionalized settings in Germany. Such knowledge is important to better understand the importance of loneliness among the oldest old residing in institutionalized settings. Furthermore, identifying the determinants may assist in addressing those institutionalized individuals at risk for high loneliness levels. This can help to promote health and successful ageing in this particularly vulnerable group.

## Methods

### Sample

Data from the survey on the quality of life and subjective well-being of very old people in North Rhine-Westphalia (NRW80+) were used. This survey was conducted in North Rhine-Westphalia, the most populous federal state of Germany, from August 2017 to February 2018. The NRW80+ is a representative sample of individuals in this federal state who are 80 years or older. Men and individuals aged 85 years and older were oversampled. Thus, sampling weights were applied in our study to account for such factors. Details about the weighting procedure are given in Chapter 8 of the methods report [4] and also by Hansen et al. [19]. In our study, we concentrated on individuals in institutionalized settings. Thus, in our analytical sample, *n* equalled 142 individuals.

### Outcome: loneliness

In accordance with prior research (e.g., [10, 24, 36, 44]), a single item measure was used to quantify loneliness. It ranges from 1 (never/almost never) to 4 (always or almost always). Prior research has shown that the UCLA loneliness scale is highly associated with such a single item measure of loneliness [33].

Following Gardiner et al. [13], for descriptive purposes we trichotomized this item into:

- not lonely if individuals responded to this single item with 1 (never lonely),
- moderately lonely if individuals responded to this single item with 2 (sometimes lonely),
- severely lonely if individuals responded to this single item with 3 or 4 (mostly lonely or almost always lonely).

Gardiner et al. [13] followed the classification scheme for loneliness developed by Valtorta et al. a few years ago [48]. This choice also eases the comparison to the results of the systematic review/meta-analysis conducted by Gardiner et al. [13].

However, for reasons of transparency, we additionally present all four loneliness levels in the results section. In multiple linear regression analysis, we used loneliness (measured continuously, i.e., from 1 to 4) as outcome measure.

### Determinants

Grounded on prior research (overview: [8]) and theoretical considerations, determinants were selected. In regression analysis, we included age, sex, marital status (single, divorced, widowed, married but separated from spouse, married), education (International Standard Classification of Education (ISCED)-97 [46] classification: low, medium or high), social network size, activity score consisting of 16 activities such as sports, volunteering, walks (from 1 to 5, higher values reflecting a higher frequency), number of technologies in use (6 items such as smartphone or internet use; score ranges from 0 to 1, with higher values reflecting a higher number of technologies in use), self-rated health ranging from 1 (very bad) to 4 (very good), functional impairment (modified Lawton and Brody instrumental activities of daily living (IADL) scale; 7 items; score ranges from 0 to 2, with higher values reflecting lower functional impairment), depressive symptoms (depression in old age scale, DIA‑S, with favorable psychometric characteristics [21, 22]; 4 items; score ranges from 0 to 4, with higher values reflecting more depressive symptoms), and cognitive impairment (DemTect [26, 28]; total score ranges from 0 to 18, with higher values corresponding to lower cognitive impairment; scores between 9 and 12 indicate mild cognitive impairment, and scores lower than 9 indicate dementia; scores from 13–18 indicate no cognitive impairment).

### Statistical analysis

First, the prevalence of loneliness was reported among all individuals aged ≥ 80 years living in institutionalized settings and also stratified by key sociodemographics (i.e., sex, age group, education and marital status). Subsequently, multiple linear regressions (with robust standard errors) were used to examine the determinants of loneliness. Full information maximum likelihood (FIML) was used to address missing values [2, 49].

## Results

### Sample characteristics

The average age was 90.2 years (SD 4.9 years, ranging from 80 to 101 years). In sum, 73.2% of the individuals were female. The prevalence of loneliness is shown in Table 1 (also stratified by age group, sex, educational level and marital status). About 56.6% of the individuals were not lonely, 25.7% of the individuals were moderately lonely and 17.8% of the individuals were severely lonely.

**Table 1:** Prevalence rates for not lonely, moderately lonely and severely lonely, also stratified by sex, age group, education and marital status

	Not lonely (%)	Moderately lonely (%)	Severely lonely (%)
Total sample	56.6 (47.0 to 65.7)	25.7 (18.6 to 34.4)	17.8 (11.5 to 26.5)
*Sex*			
Men	46.7 (27.7 to 66.7)	43.1 (24.8 to 63.4)	10.3 (3.0 to 29.7)
Women	58.7 (47.9 to 68.8)	21.9 (14.6 to 31.5)	19.4 (12.1 to 29.6)
*Age group*			
80–84 years	64.7 (36.8 to 85.2)	16.8 (4.5 to 46.8)	18.5 (5.3 to 48.3)
85–89 years	55.4 (39.4 to 70.4)	23.7 (13.2 to 38.8)	20.9 (10.7 to 38.8)
90 years and over	52.6 (40.6 to 64.4)	32.9 (22.7 to 45.1)	14.4 (7.5 to 26.2)
*Education*			
Low education	57.4 (41.4 to 72.0)	27.4 (16.1 to 42.7)	15.2 (6.9 to 30.1)
Medium education	57.4 (42.3 to 71.2)	22.0 (12.2 to 36.5)	20.6 (10.6 to 36.2)
High education	44.1 (20.0 to 71.4)	28.6 (10.4 to 58.0)	27.3 (9.5 to 57.3)
*Marital status*			
Single; divorced; widowed; married, but separated from spouse	57.4 (47.3 to 66.9)	28.3 (20.4 to 37.9)	14.3 (8.6 to 22.8)
Married	51.1 (22.3 to 79.2)	7.9 (1.9 to 27.8)	41.0 (15.5 to 72.5)

Prevalence rates are displayed, 95% confidence interval (*CI*) is shown in parentheses

When using all 4 loneliness levels, i.e., additionally distinguishing between mostly lonely and (almost) always lonely, 12.7% (95% confidence interval [CI]: 7.3–21.2%) of the individuals belonged to the mostly lonely group and 5.0% (95% CI: 2.4–10.4%) of the individuals belonged to the (almost) always lonely group.

### Regression analysis

The findings of multiple linear regressions are given in Table 2, the R^2^ value was 0.35. Mean average variation inflation factor (VIF) was 1.5 (highest VIF was 2.1) indicating that multicollinearity is not a threat.

**Table 2:** Determinants of loneliness. Results of multiple linear regressions

Independent variables	Loneliness
Sex: women (Ref.: men)	0.16
	(0.14)
Age	0.003
	(0.01)
Marital status: married, living together with spouse (Ref.: married, living separated from spouse, widowed, divorced, single)	0.48*
	(0.21)
Education: – medium (Ref.: low)	0.17
	(0.14)
– high	0.46*
	(0.20)
Size of the social network	−0.02*
	(0.01)
Activities: frequency	0.04
	(0.13)
Technology usage: number	−1.67^+^
	(0.98)
Self-rated health (ranging from 1 = very bad to 4 = very good)	−0.25*
	(0.12)
Functional impairment	−0.004
	(0.11)
Depressive symptoms	0.25***
	(0.05)
Cognitive impairment: – Presence of mild cognitive impairment (Ref.: absence of mild cognitive impairment/dementia)	−0.30
	(0.21)
– Dementia	−0.10
	(0.22)
Constant	1.42
	(1.14)
R^2^	0.35
Observations	142

Unstandardized beta-coefficients are displayed, robust standard errors in parentheses

***p < 0.001, **p < 0.01, *p < 0.05, +*p* < 0.10

Regressions showed that higher loneliness is associated with being married (β = 0.48, *p* < 0.05), high education (compared to low education, β = 0.46, *p* < 0.05), a small social network size (β = −0.02, *p* < 0.05), poor self-rated health (β = −0.25, *p* < 0.05), and more depressive symptoms (β = 0.25, *p* < 0.001). Moreover, there was a marginal significant association between a higher number of technology usage and lower loneliness (β = 1.67, *p* = 0.09). Sex, age, the frequency of activities, functional impairment and MCI/dementia were not associated with loneliness.

## Discussion

Nearly 50% of individuals were moderately or severely lonely in our study. This prevalence is in marked contrast to individuals living in private homes where about 25% of individuals were moderately or severely lonely. For reasons of comparison, it may be also worth noting that among noninstitutionalized individuals in the NRW80+, 76.3% of the individuals were not lonely, 19.0% of the individuals were moderately lonely and 4.7% of the individuals were severely lonely.

Prior knowledge was mainly restricted to qualitative studies (overview: [39]) or mostly did not exclusively focus on the oldest old [13]. Moreover, quantitative studies were missing regarding the prevalence and determinants of loneliness in oldest old residents of nursing or old age homes in Germany. Our current study thus adds first quantitative evidence regarding loneliness experiences among the oldest old in Germany (North Rhine-Westphalia).

The rather high prevalence rates of loneliness among the oldest old extend our current knowledge mainly based on prior findings from the general adult population in Germany [16]. A prior systematic review and meta-analysis [13] investigating the prevalence of loneliness among older people living in residential and nursing care home settings also reported very high average prevalence rates (i.e., moderate loneliness 61%; severe loneliness 35%). Potential reasons for such prevalence rates include that living in institutionalized settings (as opposed to living in a private home) can disconnect the affected individuals from the family and close friends [5, 20]. Moreover, the identified prevalence rates can be explained by a wide range of physical and mental conditions associated with highest age [12]. Furthermore, the quality and kind of care and support received by the oldest old in the nursing homes might be lower compared with the quality of community care, which may also contribute to these frequencies.

The association between being married and higher loneliness scores is very counterintuitive and in contrast to a systematic review mainly based on data from community-dwelling samples [8]. We assume that our findings can be explained by the fact that institutionalized individuals may extremely miss their spouses. A prior qualitative study [23] in Norwegian nursing homes may support this assumption. The aim of this former study was to explore the perceptions of sadness among older people residing in nursing homes (in Rogaland County, Norway in 2012/2013). For example, one male individual stated: “We have been married for ages. Moving into nursing home was like getting a divorce. It was very sad, even though she visits me every day” (page 3) [23]. The spatial separation or reduced frequency of contact could increase the feeling of loneliness among institutionalized individuals [11, 47]. Additionally, unmarried individuals might find it easier to make new contacts due to less emotional family burden (e.g., form new relationships with members of staff or other residents [31]).

Individuals with high education showed higher loneliness scores compared to individuals with low education in our study. This link may be explained by the fact that the former group may have had quite frequent social activities prior to admission to nursing or old age home [32]. Thus, they may perceive a higher discrepancy between actual and desired social contacts (discrepancy model of loneliness). They also may miss particularly these social activities in institutionalized settings and may thus feel lonely [5].

An association between a smaller network size and higher loneliness scores was identified in our current study, which is well in line with prior research (e.g., [9]). Such an association confirms the theory of the need to belong introduced by Baumeister and Leary [3], which emphasizes the basic human need to belong to someone. For example, a prior study also showed that a high frequency of contact with sons, daughters and grandchildren is associated with lower social loneliness levels among residents in nursing homes [9]. A prior qualitative study showed that a sense of belonging to the family, friends and the staff from the nursing home may prevent loneliness among individuals living in Norwegian nursing homes [42].

Our study showed an association between poor self-rated health, more depressive symptoms and higher loneliness scores. This is in accordance with prior research among institutionalized individuals in very late life (e.g., [24]) and can be explained by, among other things, the associated impairments in their life. More precisely, health difficulties could make it more difficult to have close relationships with peers and staff in nursing homes [43]. For example, health impairments may reduce the noncare time and noncare time can contribute to close relationships with staff in nursing homes [43].

We would like to highlight some strengths and limitations of this current work. First, as one of the first studies [30, 36], we exclusively used data from oldest old institutionalized individuals (i.e., aged 80 years and over). Moreover, it should be noted that data from a representative sample were used. Additionally, established tools were used to quantify the variables of interest. Our study has a cross-sectional design, which limits the possibility to clarify the causal link. Thus, upcoming research in this area is required. Furthermore, upcoming research could focus on mediating and moderating factors (e.g., [53]), which is beyond the scope of our current work. Furthermore, our determinants were somewhat limited in our study. For example, personality factors (e.g., extraversion) could also be associated with loneliness [6] among institutionalized individuals. This should be examined in future studies.

In conclusion, a significant proportion of the oldest old institutionalized individuals reported moderate or severe loneliness, which underpins the relevance of this topic. Knowing the determinants of loneliness may help to address institutionalized individuals aged 80 years and over at risk.

A prior systematic review suggested that several interventions (e.g., reminiscence therapy or laughter therapy) can assist in reducing loneliness scores among older adults living in long-term care facilities [41]. With respect to future research, this should be further explored.

## Declarations

For this article no studies with human participants or animals were performed by any of the authors. All studies mentioned were in accordance with the ethical standards indicated in each case.
